# The world report on hearing, 2021

**DOI:** 10.2471/BLT.21.285643

**Published:** 2021-04-01

**Authors:** Shelly Chadha, Kaloyan Kamenov, Alarcos Cieza

**Affiliations:** a Sensory Functions, Disability and Rehabilitation Unit, Department for Noncommunicable Diseases, World Health Organization, Avenue Appia 20, 1211 Geneva 27, Switzerland.

In 2017, the World Health Assembly drew attention to the rising global prevalence of hearing loss. The Health Assembly called upon the World Health Organization to develop an evidence-based world report on hearing that provides guidance to Member States for improving access to ear- and hearing-care services. The report, developed through a multistakeholder consultative process, and published in March this year, reveals that by 2050 nearly 2.5 billion people will be living with some degree of hearing loss; of these, at least 700 million will require rehabilitation services.^1^^,^^2^ Failure to act will be costly both in terms of the health and well-being of those affected, and the financial losses arising from their exclusion from communication, education and employment.

The report highlights why investing in preventing and addressing hearing loss is warranted at this time. First, across the life course, hearing loss can be prevented. Over a billion young individuals are at risk of avoidable hearing loss associated with recreational use of personal devices^3^ and about 200 million have chronic ear infections^4^ that can be prevented or treated. Second, effective and cost-effective solutions for the identification and rehabilitation of hearing loss are already benefiting millions of people at all stages of life. Combining the power of these interventions with public health strategies will ensure that those interventions can reach all in need.^1^ Third, in 2020 nearly 1 trillion international dollars were lost due to unaddressed hearing loss globally.^5^ With the current prevalence, unless action is taken, this amount may grow in coming decades. Fourth, an additional annual investment of 1.33 United States dollars (US$) per capita by governments can increase coverage of ear and hearing care services up to 90%, benefiting 1.3 billion people and bringing a return of around US$ 16 for every dollar invested over the next 10 years.^1^

The report further elucidates the huge gap in services for ear and hearing care in all parts of the world. For example, there is an estimated gap of 83% between the need for and access to services for such care – taking hearing aid use as a tracer indicator.^6^ The reasons for this gap are manifold and include the lack of accurate information and stigmatizing mindsets surrounding ear diseases and hearing loss.^7^^,^^8^ Even among health-care providers, knowledge relevant to prevention, early identification and management of hearing loss and ear diseases is commonly lacking, thereby hampering their ability to provide the care required.^8^^–^^10^ Moreover, in most countries, especially low- and middle-income, ear and hearing care is not integrated into health systems, which commonly lack the capacity to deliver the required services at primary and secondary levels, where they are most needed. The most glaring gap in health system capacity is in human resources.^11^ Among low-income countries surveyed, 78% (14/18) have fewer than one ear, nose and throat specialist per million population and 93% (14/15) have fewer than one audiologist per million. Even in countries with relatively high proportions of professionals in the field of ear and hearing care, inequitable distribution and other factors can limit access. This lack of access poses challenges for people in need of care and places unreasonable demands on the cadres providing these services.^11^

These challenges can be overcome through strategic government-led planning that integrates people-centred ear and hearing care within national health plans for universal health coverage (UHC). Care services can be implemented through adoption of established public health approaches such as task-sharing and telemedicine. Countries can deliver such people-centred care throughout the life course by ensuring access to evidence-based interventions that are delivered through a strengthened health system. The *World report on hearing* proposes such a package of interventions^1^ that countries should consider when developing their national health plans or policies for UHC. This package, called H.E.A.R.I.N.G., includes hearing screening and intervention; ear disease prevention and management; access to technologies; rehabilitation services; improved communication; noise reduction; and greater community engagement. Identified through a consultative process based on evidence of their effectiveness, the first four concepts must be integrated and delivered through strengthened health systems. Countries should determine which interventions best suit their needs by conducting an evidence-based consultative prioritization exercise.

Without integrated people-centred ear and hearing care, governments risk depriving their citizens of the right to achieve the highest possible standard of health, functioning and well-being; and of the possibility of communicating optimally with others. The report on hearing presents a call to action, inviting Member States to work for the needs of all those who need ear and hearing care and to achieve the target of a 20% relative increase in effective coverage of such services by 2030.
